# To Treat or Not to Treat Poorly Differentiated Adenocarcinoma of Unknown Origin

**DOI:** 10.7759/cureus.25216

**Published:** 2022-05-22

**Authors:** Tara Alleyasin, Sandra Abadir, Allison Holley, Darby Sider

**Affiliations:** 1 Internal Medicine, Florida Atlantic University Charles E. Schmidt College of Medicine, Boca Raton, USA; 2 Internal Medicine, Ross University School of Medicine, Los Angeles, USA; 3 Internal Medicine, Cleveland Clinic, Weston, USA

**Keywords:** hospice and palliative care, hospice, platinum based, poorly differentiated, cancer of unknown origin, adenocarcinoma

## Abstract

This case illustrates that although advances have been made with diagnosis and treatment of adenocarcinoma of unknown origin with targeted therapy, more research needs to be done on poorly differentiated adenocarcinoma that initially presents with extensive metastases. In this patient’s case, it was beneficial and ethical to reduce the toxicity and emotional burden and thus limit further investigation into her adenocarcinoma. However, it is imperative to recognize that only a small subset of adenocarcinoma of unknown origin are responsive to current therapies and more research is required for the many cases that present with poorly differentiated adenocarcinoma and widespread metastasis.

## Introduction

Cancer of unknown origin (CUO) is considered the eighth most common malignancy worldwide with adenocarcinoma accounting for 70% of these neoplasms [1,2]. Presenting symptoms depend on the sites of metastasis, and in 75-80% of the cases, the primary tumor remains unidentified even after extensive workup [2]. Due to the widespread metastasis at time of presentation, the prognosis is generally poor with a median survival rate of less than a year [1]. Despite the availability of various treatment protocols, this case demonstrates that there is no specific protocol for the management and treatment of poorly differentiated adenocarcinoma of unknown origin, thus making withholding treatment the best option [1].

## Case presentation

This case is about a 34-year old woman who was admitted and treated for abdominal pain. The pain was believed to be caused by “alcohol-induced pancreatitis”. At that time her lipase was mildly elevated at 145 U/L and her D-dimer was 19,420 ng/ml. A computed tomography (CT) chest with contrast was negative for a pulmonary embolism but had findings suggestive of acute pancreatitis. The patient further admitted to recent binge drinking. Within a week, she was admitted two more times with the same abdominal pain and CT findings. The patient was stabilized and discharged once her symptoms resolved. Prior to these three episodes, the patient never complained of similar symptoms. Her parents denied any previous illnesses including heart conditions. 

**Figure 1:**
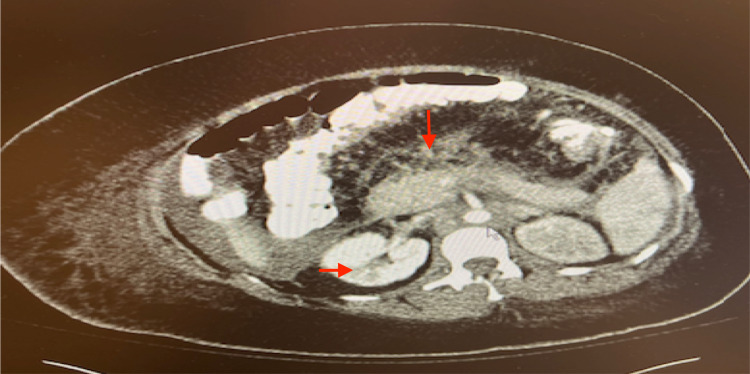
Pancreatitis and renal infarction on CT scan of abdomen/pelvis with IV contrast Computed Tomography (CT) showing severe left hydronephrosis without discrete ureteral calculus. There is also left renal cortical hypo-enhancement, which is suggestive of pyelonephritis and acute pancreatitis (vertical red arrow) but no pancreatic necrosis or peri-pancreatic fluid. Bilateral kidneys were significant for renal infarctions (horizontal arrow).

Eight days later, she was brought to the emergency department with sudden loss of consciousness, aphasia, and right-sided weakness. At this admission, she was found to have acute thrombosis of the left middle cerebral artery on a CT scan of the brain. A follow-up magnetic resonance imaging (MRI) of the brain demonstrated punctate areas of acute ischemia bilaterally in the supratentorial and infratentorial regions. She continued to have aphasia, was not following commands, and she was not oriented to person, place, or time. Doppler ultrasound (US) demonstrated several deep vein thrombosis (DVT) in all four extremities. Repeat CT scan of the abdomen and pelvis showed pancreatitis and wedge-shaped hypodensities in the spleen and kidneys concerning for infarction (Figure 1).

An echocardiogram revealed a 0.9 cm x 0.6 cm mass in the atrial side of the posterior mitral leaflet. Attempts to find more about her family history were unsuccessful as she was adopted and her adoptive parents lacked information about her biological parents. Additionally, she was found to have ascites and pleural effusion. Fluid analysis was positive for metastatic poorly differentiated adenocarcinoma. Immunostaining expressed moc-31, Ber-EP4, CK7, CK20 (subset), CDX2, lack GATA3 and PAX8. CA19-9 and CA-125 were elevated at 21,740 U/ml and 364 U/ml respectively. Her admission was further complicated by spontaneous bacterial peritonitis, severe hydronephrosis, urinary tract infection, and heparin-induced thrombocytopenia. Given the aggressive nature of the metastatic adenocarcinoma, the oncologists recommended against further treatment. Further steps were communicated with the patient who remained incoherent. Her parents decided that she should go home with them so that her two children can spend some time with her. Patient was discharged to hospice and shortly after passed away. Further ordered genetic testing and investigations to further diagnose the primary cancer were halted.

## Discussion

Modes of investigation to attempt to search for the primary tumor include a whole-body positron emission tomography-computed tomography (PET/CT), histological findings, immunohistochemistry, tumor markers, mammography and vaginal US in women, prostate US in men, CT scans and MRIs [1,3]. While treatment plans are formed based on individual patients and their staging and risk assessment, most chemotherapy regimens contain platinum [4]. The use of therapy containing platinum has been effective in patients who had a prodrome phase of symptoms and thus the progression of their metastasis was less severe [3,4]. 

Poor prognosis makes the decision for extensive workup and treatment controversial due to potential medication toxicity. Hence, patients with favorable outcomes ought to be identified to further investigate and develop effective treatment plans [3]. A minor subtype of patients fall under the favorable outcome category; these are patients with differentiated and chemosensitive tumors [4]. Our patient presented with abdominal pain only a few weeks before she experienced the full effect of the metastasized cancer and was ultimately determined that hospice would be the best choice. The targeted treatment for CUO is based on the gene expression profiling of the tumor tissue [1]. Platinum-based empiric therapy is recommended and the first line of therapy for CUO based on the profiling. However, patients with poor prognosis are less likely to benefit [1]. Prognosis in CUO can be predicted by several factors such as lactate dehydrogenase (LDH) and albumin levels, performance status, and metastasis [4]. However, there has been very limited research on targeted therapy along with empiric therapy for CUO with rapid advancement in their cancer [4]. It is imperative to recognize that only a small subset of adenocarcinoma of unknown origin are responsive to therapy and more research is required for the cases that present with poorly widespread metastasis of poorly CUO.

## Conclusions

This case illustrates that although advances have been made with diagnosis and treatment of adenocarcinoma of unknown origin with targeted therapy as well as platinum-based chemotherapy, more research needs to be done on poorly differentiated adenocarcinoma that initially presents with extensive metastases. In this patient’s case, it was beneficial and ethical to reduce the toxicity and emotional burden for the patient and her family and thus limit further investigation into her adenocarcinoma. However, it is imperative to recognize that only a small subset of adenocarcinoma of unknown origin are responsive to current therapies and more research is required for the many cases that present with poorly differentiated adenocarcinoma and widespread metastasis.
